# Perioperative Outcomes in Orthopedic Surgery

**DOI:** 10.1155/2015/648284

**Published:** 2015-05-20

**Authors:** Stavros G. Memtsoudis, Vassilios I. Vougioukas, Ottokar Stundner, Lazaros A. Poultsides

**Affiliations:** ^1^Hospital for Special Surgery, 535 East 70th Street, New York, NY 10021, USA; ^2^University of Freiburg, 79110 Freiburg im Breisgau, Germany; ^3^Paracelsus Medical University, 5020 Salzburg, Austria

With millions of surgical procedures being performed worldwide annually, clinicians, researchers, patients, and administrators increasingly pursue understanding factors, which affect and determine perioperative outcomes. The last decades have been associated with extensive research and subsequent increased number of publications in the field of perioperative outcomes in orthopaedic surgery. The population is progressively involved in recreational and athletic activities increasing thus the prevalence of sport-related soft-tissue and bone injuries. Furthermore, the population is aging and the demand for joint replacement and spine surgery is increasing annually, raising inevitably the absolute numbers of surgery related complications such as infection, mechanical failure, and delayed union. Finally, the adoption of new technology and the introduction of minimally invasive surgical techniques still remain a controversial topic on whether evidence-based medicine has justified the risk of their use. Hence, it is important to critically evaluate the perioperative outcomes of modern orthopaedic surgery and further investigate whether a change in the perioperative management of the orthopaedic patient is warranted. Although the selected papers for this special issue are not an exhaustive representation of the area of perioperative outcomes in orthopaedic surgery, they represent an excellent panel for approaching and addressing this challenge. Without doubt they will provide significant knowledge to the readers, simulating further investigation and research and possible improvement of the current surgical technique and overall perioperative management of the orthopaedic patient.

The special issue contains six papers; of these, three papers are related to spine surgery and more specifically to fusion rates of various disc substitutes and outcomes and complications of minimally invasive surgery. One paper presents short-term outcomes of surgical reconstruction of chronic functional ankle instability. Another experimental study provides data that support the combined use of flexible intramedullary nailing and Ilizarov external fixation for bone repair enhancement. Finally, the last paper covers criteria for patient risk stratification with regard to the development of surgical site infection following total hip arthroplasty.

In the paper entitled “PEEK Cages versus PMMA Spacers in Anterior Cervical Discectomy: Comparison of Fusion, Subsidence, Sagittal Alignment, and Clinical Outcome with a Minimum 1-Year Follow-Up,” authors compared radiographic and clinical outcomes after anterior cervical discectomy in patients with cervical degenerative disc disease using PEEK cages or PMMA spacers with a minimum 1-year follow-up. They found that the substitute groups showed differing fusion rates. However, clinical outcomes appeared to be generally not correlated with fusion status or subsidence. Authors concluded that they could not specify a superior disc substitute for anterior cervical discectomy.

In the paper “Minimally Invasive Technique for PMMA Augmentation of Fenestrated Screws” authors described the minimally invasive technique, as well as its safety and efficacy, for cement augmentation of cannulated and fenestrated screws using an injection cannula. The presented minimally invasive cement augmentation technique using an injection cannula was proven to facilitate convenient and safe cement delivery through polyaxial cannulated and fenestrated screws during minimally invasive screw-rod spondylodesis.

In the paper “Accidental Durotomy in Minimally Invasive Transforaminal Lumbar Interbody Fusion: Frequency, Risk Factors, and Management,” authors' purpose was to assess the frequency, risk factors, and management of accidental durotomy in minimally invasive transforaminal lumbar interbody fusion. Their results showed that the frequency of accidental durotomies in MIS TLIF is low, with overweight being a risk factor. They concluded that the minimally invasive approach seems to minimize durotomy-associated complications (CSF leakage, pseudomeningocele) because of the limited dead space in the soft tissue.

In the paper “Surgical Reconstruction with the Remnant Ligament Improves Joint Position Sense as well as Functional Ankle Instability: A 1-Year Follow-Up Study,” they assessed functional improvement of chronic ankle instability after surgical reconstruction using the remnant ligament. This study showed that surgical reconstruction using the remnant ligament was effective not only for improving mechanical retensioning but also for ameliorating joint position sense and functional ankle instability.

In the paper “Bone Healing by Using Ilizarov External Fixation Combined with Flexible Intramedullary Nailing versus Ilizarov External Fixation Alone in the Repair of Tibial Shaft Fractures: Experimental Study,” authors conducted an experimental study utilizing an open tibial shaft fracture canine model aiming to study the radiographic and histological outcomes of flexible intramedullary nailing (FIN) combined with Ilizarov external fixation (IEF) versus Ilizarov external fixation alone. Authors concluded that the combination of the Ilizarov apparatus and FIN accelerates bone repair and augments stabilization of tibial shaft fractures as compared with the use of the Ilizarov fixation alone.

Finally, in the paper “Patient, Surgery, and Hospital Related Risk Factors for Surgical Site Infections following Total Hip Arthroplasty” authors conducted an extensive review of the literature on reported patient, surgery, and hospital related risk factors for SSI after THA. This review can facilitate surgeons to identify patients at risk for infection and administrators to adopt preventive systems based strategies to reduce the overall risk and therefore the subsequent multifaceted burden of periprosthetic joint infections.

